# Unmasking the Clever Hans effect in AI models: shortcut learning, spurious correlations, and the path toward robust intelligence

**DOI:** 10.3389/frai.2025.1692454

**Published:** 2026-01-09

**Authors:** Abhay Kumar Pathak, Manjari Gupta, Garima Jain

**Affiliations:** 1 Department of Computer Science, Institute of Science, Banaras Hindu University, Varanasi, India; 2 MIRNOW, BIONEST, Banaras Hindu University, Varanasi, India

**Keywords:** Clever Hans effect, spurious correlation, shortcut learning, model robustness, responsible AI

## Abstract

The Clever Hans (CH) effect is a historical analogy of a horse solving mathematical problems based on some cues, representing a critical failure in artificial intelligence (AI) systems, where models achieve higher performance by utilizing spurious correlations and artifacts presented in the datasets rather than relying on causal relationships or task-related features. This effect or phenomenon is prevalent across multiple domains of AI such as computer vision, natural language processing, medical imaging, and reinforcement learning. This review examines the Clever Hans effect, the conceptual foundation of spurious correlations, and current evaluation methods that obscure such behavior. We further survey state-of-the-art detection and mitigation strategies, focusing on both model-centric and data-centric techniques. Building on these insights, we propose a roadmap for robust AI development, which includes standard benchmarking, causal integration, human-in-the-loop auditing, and transparent policy frameworks. This study underscores that addressing the Clever Hans effect is not only necessary for technical robustness but also for the ethical and responsible deployment of AI systems in real-world, high-stakes environments.

## 1 Introduction

The evolution of artificial intelligence (AI) has been marked by a series of transformations that have shaped both technological advancement and its applications in society. In its initial stage, AI relied on symbolic learning and rule-based methods for mundane tasks such as spam detection, recommendation systems, classification, and advertising. As the field has progressed, it has evolved through multiple stages encompassing machine learning (ML), deep learning (DL), large language models (LLMs), and advanced automation, enabling increasingly complex applications (Bommasani et al., 2022). This reflects a clear shift from task-specific algorithms to generalized models capable of solving cross-domain problems. As AI matured, its utility significantly expanded, enabling integration into domains such as finance, healthcare, education, agriculture, and law (Rane et al., 2024). In daily life, AI performs many mundane tasks with high reliability, such as virtual assistants, SIRI and Alexa, which utilize natural language processing for understanding speech in a real-time environment (Hassija et al., 2023). AI demonstrates significant capabilities through both supervised and unsupervised learning paradigms. In supervised ML, models perform precise predictive tasks, such as disease prediction, financial forecasting, and weather prediction, by learning from labeled data, which provides context for the data points (LeCun et al., 2015). In contrast, unsupervised learning tries to find hidden patterns and structures without using labels in data, which is important for tasks such as anomaly detection and clustering across cross-domain problems. AI-powered systems for facial recognition in traffic cameras, recommendation systems on social media platforms, fraud detection in online banking, and threat assessment in networking devices are prime examples that illustrate the development of AI over time (Mikhaylov et al., 2018). Healthcare has been transformed by AI-driven assisted diagnostics, personalized care, patient monitoring, and clinical decision support systems, significantly improving the speed, accuracy, and efficiency of patient care (Topol, 2019; Singh et al., 2025; Pathak et al., 2023; Pathak et al., 2024).

Despite remarkable advances, AI models remain vulnerable to systematic biases arising from both the architecture of the model and the distribution of data. A well-known issue is data bias, which happens when training data reflect imbalanced, incomplete, or non-representative instances of the real-world population (Mehrabi et al., 2021). Such biases manifest during the training phase of the model, often leading models to rely on unintended spurious correlations or features—statistical associations that do not reflect meaningful or causal relationships (Geirhos et al., 2020). For example, vision models trained on ImageNet may learn background textures correlated with object classes rather than the objects themselves. Similarly, models trained on medical images have been shown to rely on confounding factors such as scanner type, hospital or facility identifiers, machine-specific characteristics, or embedded imaging artifacts, instead of the true clinical features that the models are intended to learn. These scenarios degrade model performance when deployed in real-world settings across different institutions (Zech et al., 2018). Convolutional Neural Networks have been shown to rely on the presence of rulers or variations in skin tone when predicting cancer, rather than focusing on the morphological features of the lesion itself (Winkler et al., 2019).

Data biases are not limited to image processing; in natural language processing, models often rely on unintended syntactic patterns, question templates, or lexical cues while ignoring the semantic understanding of the text (Delaney et al., 2023). In a similar vein, large language models (LLMs) display shortcut behavior by mimicking prompt formats and token distributions, which can lead to misleading outputs or hallucinated responses when these patterns change (Lin et al., 2022).

These biases are also present in speech and sensor-based domains, where AI models are often latched onto the frequency signatures of the microphone, the acoustics of the surroundings, or the metadata of the device that are unintentionally related to the target label, making them highly vulnerable to environmental changes (Martin and Wright, 2023).

In time series models, these scenarios often occur when models trained on clinical data identify timestamps and monitor brand identifiers as important features. These features do not reflect the actual physiology of individuals but can still influence outcomes (Harutyunyan et al., 2019).

This results in the degradation of AI model robustness across domain-specific tasks, where models may show high performance during training by relying on spurious correlations and cues but fail under different circumstances (Maheronnaghsh and Alvanagh, 2025).

Moreover, explainable AI methods such as Grad-CAM, SHAP, and LIME often fail to reveal the true behaviors of models trained on spurious features. This highlights a phenomenon known as the Clever Hans (CH) effect, where an AI model appears intelligent but relies on unintended features or misleading cues (Lapuschkin et al., 2019).

Several factors are responsible for the emergence of the CH effect in AI systems: (1) Data artifacts and embedded biases, which often serve as shortcuts for models, allowing them to achieve unusually high performance during training. Examples include scanner-specific information and metadata in medical imaging, background details in image datasets, or emoji frequency patterns that can mislead models in sentiment analysis (Zech et al., 2018). (2) Absence of causal supervision, which causes models to overfit using superficial correlations instead of learning task-relevant and invariant features (Sagawa et al., 2019). (3) Imbalanced or non-representative datasets, which introduce hidden confounders, such as socio-economic proxies or demographic imbalances, shifting the attention of the model toward spurious cues. (4) Insufficient evaluation pipelines, which generally rely on independent and identically distributed splits that preserve the same biases across training and testing sets, thereby masking shortcut reliance. (5) Lack of robust interpretability tools, which makes it difficult to detect when a model is utilizing non-causal cues; explainable AI methods often produce plausible but misleading attributions (Lapuschkin et al., 2019). Furthermore, factors such as reward hacking in reinforcement learning, dataset leakage, and the absence of out-of-distribution validation are also reasons for the emergence of the CH effect in modern AI models.

As AI systems are now an integral part of our daily life, spanning healthcare, finance, autonomous systems, and decision-making, there is a growing need for assurance that models are not only high-performing but also robust, generalizable, and interpretable. This is often overlooked during standard training and evaluation, resulting in models that perform well under controlled benchmarks but fail during deployment when distribution shifts occur in real-world scenarios. Such a nature of models allows them to appear intelligent while relying on unintended non-semantic or non-causal signals, raising concerns about their reliability, trustworthiness, and fairness. Given these scenarios, there is an urgent need to understand how shortcut learning emerges, how it can be detected and diagnosed, and what mitigation strategies are more effective when dealing with it.

In this review, we provide a comprehensive survey of AI model vulnerabilities arising from the Clever Hans effect—also referred to as spurious correlations and shortcut learning. We critically examine the behavioral effects across domains, such as natural language processing, medical imaging, computer vision, and speech processing, where they undermine model generalization and robustness. Furthermore, we identify and evaluate the most effective detection and mitigation methods developed in recent years, categorizing them into model-centric and data-centric approaches. Finally, we present a synthesis of empirical findings, benchmark tools, and algorithmic approaches—such as invariant risk minimization (IRM), counterfactual data augmentation, and slice-aware evaluation—that can guide the development of more interpretable, transparent, and reliable AI models for high-stakes real-world environments.

## 2 Conceptual foundations of the Clever Hans effect

**Figure 1:**
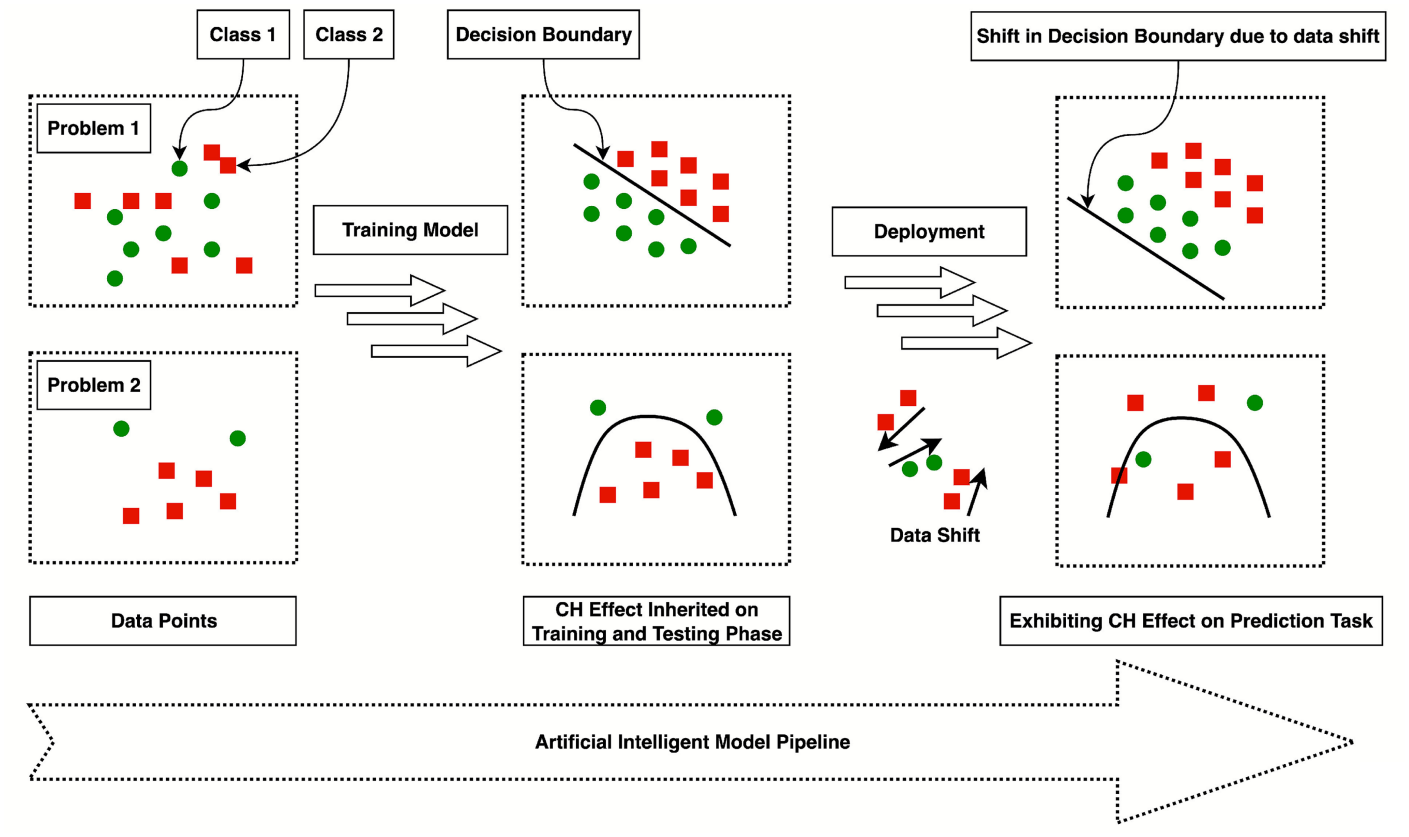
A classical example of the Clever Hans effect.

The Clever Hans effect, presented in Figure 1, takes its name from a horse in early 20th-century Germany that appeared to solve arithmetic tasks. Upon further investigation, psychologist Oskar Pfungst found that Hans was not solving actual problems mathematically but was instead responding to subtle, unintentional cues from his handler, such as changes in expression and posture, during public demonstrations (Pfungst, 2025). This historical incident serves as a strong analogy in AI, where models appear to perform complex tasks but actually exploit irrelevant or unintended signals present in the data (Lapuschkin et al., 2019). The term is used to warn against interpreting high-performing models as showing genuine understanding or reasoning. The analogy is apt because, like the horse, AI systems lack self-awareness and cannot separate causally relevant features from spurious ones without human interventions and validation (Madsen et al., 2022). Therefore, the Clever Hans effect has become a diagnostic metaphor in research on model explainability, robustness, and trustworthy AI (Hooker et al., 2019).

The Clever Hans effect in AI can be formalized using the concept of spurious correlations and shortcut learning in supervised learning models.

Suppose a model $f_{\theta }(x)$ is trained to approximate a target y=f(x) using empirical risk minimization:


$$\hat{\theta }=\arg\min_{\theta }\;E_{(x,y)\sim D_{\text{Train}}}[ℒ(f_{\theta }(x),y)].$$

However, if the training distribution $D_{\text{Train}}$ contains spurious features z⊂x that correlate with y , the model may minimize the loss by learning:


$$f_{\theta }(x)=g(z).$$

Where, g is the function of spuriously correlated variables rather than the true causal features.

Formally if,


$$\mathbb{P}(y|x_{\text{casual}})\neq \mathbb{P}(y|z).$$

But ℙ(y∣z)≈ℙ(y∣x) on $D_{\text{Train}}$ than $f_{\theta }$ exhibits the shortcut behavior relying on z rather then causal feature set $x_{\text{causal}}$ .

This phenomenon leads to distributional vulnerability, where under a shifted distribution $D_{\text{Test}}$ such that ℙ(y∣z) no longer holds and the performance of the model deteriorates.


$$\text{Generalization}\;\mathrm{Gap}=L_{\text{Test}}-L_{\text{Train}}\gg 0.$$

## 3 Manifestation of the Clever Hans effect

The manifestation of the Clever Hans effect is summarized in Table 1, highlighting its pervasiveness across both core and emerging AI application domains. In each diverse domain, models have been shown to exploit unintentional spurious features. For example, computer vision models exploit background textures, medical imaging models are influenced by hospital identifiers and scanner properties, large language models pick up on prompt patterns, and IoT systems exploit sensor-specific noise. These results show that while models may achieve high performance on benchmark datasets, they often fail to generalize under domain shifts or adapt to new test environments or adversarial conditions. The problem affects not only natural language processing, medical imaging, and textual analysis but also extends to safety-critical systems, cybersecurity, and finance. The recurring nature of the problem across domains emphasizes that it is not domain-specific, but a fundamental issue inherent to how AI systems are trained, evaluated, and deployed.

**Table 1:** A systematic mapping of shortcut learning behaviors and the Clever Hans effect across diverse artificial intelligence domains: evidence of spurious correlations, generalization failures, and contextual biases in model predictions.

AI domain	Subdomain/task	Spurious feature/shortcut	Observed problem	References
Computer vision	Image classification	Background, texture, watermark	Misclassification based on background or visual noise	Geirhos et al. (2020)
	Object detection	Contextual features (sky, road)	Detection triggered by scenery rather than object shape	Jamali et al. (2025)
Medical imaging	Chest X-ray classification	Hospital ID, scanner artifacts	Model overfits to source institution features	Ong Ly et al. (2024)
	Skin lesion analysis	Ruler, skin tone, lighting	Decision influenced by the presence of measurement tools or skin type	Nauta et al. (2022)
Natural language processing	Sentiment analysis	Emojis, punctuation	Ignores sentence meaning; overweights superficial symbols	Vosoughi et al. (2024)
	Question answering/VQA	Syntactic priors, question templates	Answers guessed from question form without visual/text grounding	Vosoughi et al. (2024)
Large language models	Factual QA/prompt completion	Prompt structure, token frequency	Hallucinated completions; high confidence in false answers	Peters and Chin-Yee (2025)
Speech and audio	ASR/command recognition	Background noise, mic frequency response	Fails with new devices or ambient sound patterns	Oglic et al. (2022)
	Speaker identification	Recording channel, environmental noise	Learns mic characteristics rather than vocal identity	Li et al. (2025)
Autonomous systems	Robot navigation	Floor texture, lighting from simulator	Model fails in real-world deployment	Muratore et al. (2022)
Time series/IoT	Human activity recognition	Device ID, sampling rate	Poor transfer across hardware or settings	Yamane et al. (2025)
	Vital sign monitoring	Patient location, time-of-day	Predictions tied to routine or room location instead of vitals	Harutyunyan et al. (2019)
Cybersecurity	Intrusion detection	IP rarity, uncommon ports	Novel but benign traffic flagged as malicious	Sommer and Paxson (2010)
Finance/risk analysis	Algorithmic trading	Timestamp, cyclical effects	Overfits to calendar patterns or market hours	Khandani et al. (2010)

## 4 Detection and mitigation strategies for the Clever Hans effect

### 4.1 Detection—data-centric

#### 4.1.1 Subgroup/slice performance analysis

The model’s performance on each data subgroup was analyzed and compared to its overall performance to identify discrepancies. These discrepancies across different data slices help identify specific subgroups in which the model fails, indicating the presence of spurious correlations, bias, or the Clever Hans effect. The study provided evidence of shortcut learning in chest X-ray and dermatology tasks and proposed a reduced attribute encoding pipeline as a core component of fairness evaluation. The findings demonstrate performance disparities in medical imaging models arising from shortcut artifacts (Brown et al., 2023).

#### 4.1.2 Confounder correlation checks

Non-causal confounders are variables that influence both input features and target labels. This association creates a spurious relationship between them, and models may learn shortcuts. The confounder correlation method detects and mitigates these non-causal artifacts using a statistical association pipeline (Qu et al., 2024).

#### 4.1.3 OOD test or sanity test

Out-of-distribution (OOD) testing evaluates the model’s behavior during the testing phase on data that are out-of-distribution relative to training data. This ensures that the model generalizes well to unseen data; failure to do so may indicate the presence of spurious correlations or shortcut learning. In contrast, sanity tests assess logical correctness, verify input preprocessing, and detect unreasonable predictions and errors (Mahmood et al., 2021).

### 4.2 Detection—model-centric

#### 4.2.1 Attribution/saliency maps

Deep learning models are mostly black boxes in nature, and interpreting the learned representations of intermediate layers can reveal the model’s reliance on irrelevant features or confounding regions. Attribution/saliency maps are post-hoc methods that assign importance scores to each input for a particular output class and visually highlight the input regions that contribute most to the model’s prediction (Bassi et al., 2024).

#### 4.2.2 Occlusion/ablation sensitivity maps

An effective model-centric detection strategy systematically interrogates the trained network by ablating small patches of data. The behavioral change is measured and visualized as a heatmap, where highlighted regions indicate areas crucial for predictions.

The article shows the resiliency of various emerging transformer architectures when evaluated against the spurious correlation on three benchmark datasets, highlighting the role of the self-attention mechanism through extensive ablation studies in spuriously correlated environments (Arias-Londoño and Godino-Llorente, 2024).

#### 4.2.3 Spectral relevance analysis

By clustering multiple local heatmaps, spectral relevance analysis (SpRAy) reveals global patterns and identifies shortcut cues. The study further discusses the quantification of Clever Hans traits by SpRAy and the mitigation of a model’s Clever Hans behavior (termed Un-Hans models) through a post-hoc approach called Class Artifact Compensation (ClArC). The Clever Hans effect goes undetected by standard validation methods (Bender et al., 2023; Kauffmann et al., 2025).

### 4.3 Mitigation—data-centric

#### 4.3.1 Counterfactual and contrastive testing

These methods are hypothetical tools for causal-style reasoning, in which one or a few causal factors are altered while holding everything else constant, to observe whether the model output changes or preserves the predicted class. They detect shortcuts or Clever Hans features by searching the closest counterfactuals that cause different predictions. This strategy has been applied across various domains to expose or fix spurious correlations and biases. For example, MRI classifiers trained on brain images, in which 3D conditional generative models are used to generate brain demographic counterfactuals to mitigate the impact of demographic imbalances and shortcuts. Aligning with both data-centric and model-centric strategies, training classifiers on plausible counterfactual explanations—a perturbation technique that does not alter the underlying data distribution—has been shown to improve robustness (Pombo et al., 2023).

#### 4.3.2 Data pruning and bias correction

Sample-level or feature-level pruning of redundant, noisy, or irrelevant data, as well as features that contribute to biases in model learning, helps ensure fair generalization. Data-level resampling, reweighting, and data distribution modification are employed to mitigate contextual, statistical, or demographic biases. These approaches often incorporate implicit data pruning through preprocessing steps, such as outlier removal and noise reduction (Arias-Londoño and Godino-Llorente, 2024).

### 4.4 Mitigation—model-centric

#### 4.4.1 Explainability-guided mitigation

The *post-hoc* analysis is used to examine a model’s reliance on data, architectural components, or input regions, thereby explaining which aspects the model uses to generalize its predictions. Such analyses provide insights into the black-box nature of deep neural networks. This diagnostic process helps identify and mitigate spurious associations and shortcut learning by enabling targeted corrective actions. In particular, methods such as Layer-wise Relevance Propagation (LRP), DeepLIFT, and Bayesian CNNs have been used to reveal model attention to non-lung regions, thereby highlighting the Clever Hans effect. By masking these non-lung regions using domain expertise, improved COVID-19 detection from chest X-ray images has been achieved (Arias-Londoño and Godino-Llorente, 2024).

#### 4.4.2 Feature disentanglement and representation learning

Data may contain artifacts, such as watermarks and text tags, that become entangled with causally relevant features, leading to the Clever Hans effect. Feature disentanglement aims to capture the variation of independent, semantically meaningful factors in the data within latent dimensions and to improve performance. A study showed the presence of erroneous features in medical data, including MRI and chest radiographs (CXRs), where models exhibited improved generalization on filtered pulmonary features and pre-processed MRI scans (Trivedi et al., 2022) (Table 2).

**Table 2:** Summary of detection and mitigation strategies for shortcut learning and the Clever Hans effect in AI models.

Type	Method	Technique	Strengths	Limitations	Representative studies (2020–2025)
Detection					
Data-centric	Subgroup/slice performance analysis	OOD groups; demographic strata; bias-based slices	Detects hidden bias; highlights classification performance gaps	Requires labeled subgroups; performs best with large sample sizes	Brown et al. (2023)
	Confounder correlation checks	Feature–label correlation statistical analysis	Detects *a priori* spurious signals	Correlation does not imply causation; may overlook complex relationships	Qu et al. (2024)
	OOD test or sanity test	Behavioral testing; OOD generalization	Probes invariances; model-agnostic; scalable	Requires clearly specified test cases; black-box nature	Mahmood et al. (2021)
Model-centric	Attribution/saliency maps	Grad-CAM; SHAP; LIME; LRP	Provides visual insight; model-agnostic	Sensitive to hyperparameters; inaccurate attribute methods	Bassi et al. (2024)
	Occlusion/ablation sensitivity maps	Class artifact compensation; Grad-CAM; perturbing inputs	Pinpoints critical regions	High computational cost; occlusion may introduce artifacts	Arias-Londoño and Godino-Llorente (2024)
	Spectral Relevance Analysis (SpRAy)	Combines LRP with spectral clustering; visualization techniques t-SNE, PCA	Systematic detection method; identifies subpopulations of decisions	Requires large training data; computational overhead	Bender et al. (2023)
	Unsupervised explainable diagnostics	Latent clustering; unsupervised heatmaps; BiLRP; relevance clustering; multiple anomaly models	Generalizable across domains; Early detection; label-independent	Lacks validation; high false positive rates; scalability issues; does not attribute causality	Kauffmann et al. (2025)
Mitigation					
Data-centric	Counterfactual and contrastive testing	Generating artificial data; contrastive learning	Identifies model behavior; robust	High computational cost; requires a well-defined feature space; requires supervision for realistic counterfactuals; may overlook hidden shortcuts	Pombo et al. (2023)
	Data pruning and bias correction	Removing artifacts; reducing bias in training	Addresses bias in the training dataset; generalized models	May alter datasets’ properties; requires domain knowledge	Arias-Londoño and Godino-Llorente (2024)
Model-centric	Explainability-guided mitigation	LIME; SHAP	Provides transparency into model decision-making; model-agnostic	Computationally expensive; explanation methods may lack granularity	Kauffmann et al. (2025)
	Feature disentanglement and representation learning	Feature decoupling	More general and robust model; enhances model interpretability	Complex disentangling process; may not work with high-dimensional or noisy datasets	Trivedi et al. (2022)

## 5 Discussion

The Clever Hans Effect, a prominent manifestation of shortcut learning, is increasingly recognized as a pervasive issue compromising the reliability and robustness of supervised machine learning models. Recent literature (2020–2025) emphasizes sophisticated detection and mitigation strategies, which can be systematically categorized into data-centric and model-centric methodologies.

Among data-centric detection methods, counterfactual and contrastive testing have emerged as intuitive strategies for identifying spurious correlations. This approach involves creating modified inputs by occluding irrelevant features or altering specific contextual aspects, such as backgrounds in image classification tasks or syntactic variations in text inputs (Pombo et al., 2023). Its strengths lie in its simplicity of implementation and the intuitive interpretation of results, providing direct evidence of a model’s reliance on superficial features. However, these methods require manual generation of realistic counterfactual examples, making them labor-intensive, potentially limiting scalability, and overlooking subtler hidden shortcuts.

Subgroup or slice performance analysis complements this approach by explicitly evaluating model performance on defined subsets, such as demographic strata, institutional grouping, or out-of-distribution segments. This strategy reveals hidden performance disparities and biases, reflecting a model’s reliance on spurious correlations present in training data (Wallis and Buvat, 2022). Nonetheless, subgroup methods are limited by the need for adequately labeled subgroups and sufficient sample sizes, potentially restricting applicability in real-world settings with incomplete metadata or smaller datasets.

Confounder correlation checks further enrich data-centric detection through statistical analyses of feature–label associations. These methods systematically detect *a priori* signals indicative of spurious correlations, providing preliminary diagnostic insights. However, correlation analysis inherently lacks causal grounding and may not adequately address more intricate, non-linear interactions between features and labels, thus limiting their standalone efficacy.

Model-centric detection methods, such as attribution and saliency maps—including Grad-CAM, SHAP, and LIME—provide visual insights into the decision-making processes of black-box models by highlighting influential input regions (Anders et al., 2022). Despite their intuitive appeal, these attribution methods often exhibit sensitivity to hyperparameters and may provide misleading or imprecise feature importance maps, potentially masking true shortcut behaviors.

Furthermore, occlusion and ablation sensitivity analyses systematically perturb input data to reveal the critical regions underpinning model predictions, thereby highlighting the precise reliance of models on certain input aspects (Anders et al., 2022). However, these approaches incur considerable computational overhead and risk introducing artifacts due to artificial perturbations, limiting their broader applicability.

For mitigation, data-centric methods, such as counterfactual data generation and contrastive learning, augment datasets with systematically altered examples, intentionally breaking spurious correlations (Qu et al., 2024). Despite their effectiveness, these methods require substantial computational resources and explicitly defined feature spaces, making widespread deployment challenging.

Model-centric mitigation approaches employ explainability-guided training strategies, incorporating interpretability methods such as LIME and SHAP into the training process to actively discourage shortcut usage by promoting transparency in decision-making (Kauffmann et al., 2025). Although effective in enhancing model interpretability, these methods can be computationally expensive and may lack the granularity required for fine-tuned adjustments.

These comprehensive detection and mitigation approaches, grounded in recent technical developments, highlight the need for integrative, hybrid strategies that combine intuitive diagnostic tools, careful data management, and algorithmic robustness to effectively counter the pervasive Clever Hans effect in modern AI systems.

## 6 Roadmap for robust AI development

To address the pervasive challenge of the Clever Hans effect and shortcut learning, we present a roadmap for developing AI systems that are robust, generalizable, and trustworthy. In the first step, standardized benchmarking protocols should be established to assess model performance across multiple parameters, including generalization, reliability, and robustness. These standard benchmarks should be designed to expose potential shortcuts during the initial training of the model, providing an accurate assessment of performance when deployed in real-world and out-of-distribution (OOD) situations (Hendrycks et al., 2021). By focusing on these benchmarked parameters, we ensure that AI models are trustworthy and can adjust to new patterns in unseen data while avoiding overfitting to spurious attributes. In the second step, causal integration plays a crucial role in model design. AI systems should function based on causal relationships rather than spurious attributes that are frequently present in training data. Approaches such as IRM can help enforce causal relationships across different types of datasets (Schölkopf et al., 2021). By incorporating causal reasoning into the training process of the model, we can prevent models from exploiting superficial attributes and make sure that they learn true, meaningful patterns that generalize well across different scenarios. In the third step, incorporating human-in-the-loop auditing frameworks is essential for ensuring ongoing monitoring during model training and deployment. These frameworks enable domain experts to interfere and provide real-time feedback on decisions taken by the model, ensuring that the model is not utilizing irrelevant or harmful shortcuts. Human intervention and oversight are critical for maintaining the model’s alignment with real-world goals and ethical standards, particularly in sensitive areas such as healthcare, finance, and forecasting (Amershi et al., 2019). In the fourth step, transparent policy frameworks are essential for documenting the process of decision-making of AI systems. These frameworks encourage accountability and enable organizations to interpret and justify the behavior of AI systems in decision-making. Ongoing model evaluation and active learning mechanisms should be incorporated rigorously to ensure that models remain adaptable to data shifts. These approaches should be updated to monitor performance in real-world settings, ensuring that models remain free from shortcut training and stay focused on their respective tasks.

## 7 Conclusion

The Clever Hans effect remains a critical challenge in the development of robust, reliable, and generalizable AI systems. This phenomenon is evident across computer vision, natural language processing, and medical applications, where models often exploit spurious features that correlate with the output during training and lack a causal relationship. These behaviors may perform well on in-house data but often fail to bridge the gaps when the model encounters real-world variations and distribution shifts in data. Our review distinguishes between data-centric and model-centric paradigms in terms of detection and mitigation strategies. Detection strategies, such as counterfactual testing and slice-based performance, offer different ways to reveal spurious correlations. Each approach offers distinct strengths, ranging from intuition-based visual diagnostics to statistical robustness. Mitigation strategies, including data pruning and Grad-CAM-based feature disentanglement, help guide models toward robust, intended representations. However, these approaches often require manual intervention, extensive domain knowledge, and focused infrastructure.

While previous research provides valuable insights, current methods remain inconsistent, often tailored to specific datasets, and lack rigorous cross-domain validation. A critical challenge is the absence of unified structural benchmarks and automated pipelines capable of detecting shortcut learning throughout both training and deployment. Future research should focus on principled causal representation learning, adaptive monitoring frameworks, and consistent robustness assessments that stress-test sensitivity to non-salient features. Combining these breakthroughs with ethical and regulatory frameworks will be crucial for developing AI systems that remain reliable, transparent, and trustworthy in real-world, high-stakes environments.
